# (E)-2-Methoxy-4-(3-(4-methoxyphenyl) prop-1-en-1-yl) Phenol Ameliorates LPS-Mediated Memory Impairment by Inhibition of STAT3 Pathway

**DOI:** 10.1007/s12017-017-8469-3

**Published:** 2017-10-19

**Authors:** Ji Yeon Choi, Chul Ju Hwang, Do Yeon Lee, Sun Mi Gu, Hee Pom Lee, Dong Young Choi, Ki Wan Oh, Sang-Bae Han, Jin Tae Hong

**Affiliations:** 1 College of Pharmacy and Medical Research Center, Chungbuk National University, 194-31 Osongsaemgmyeong 1-ro, Osong-eup, Heungdeok-gu, Cheongju, Chungbuk 28160 Republic of Korea; 2 College of Pharmacy, Yeungnam University, 280, Daehak-ro, Gyeongsan, Gyeongbuk 38541 South Korea

**Keywords:** Alzheimer’s disease, Neuroinflammation, Amyloidogenesis, Signal transducer and activator of transcription 3

## Abstract

Alzheimer’s disease (AD) is pathologically characterized by an excessive accumulation of amyloid-beta (Aβ) fibrils within the brain. We tested the anti-inflammatory and anti-amyloidogenic effects of (E)-2-methoxy-4-(3-(4-methoxyphenyl) prop-1-en-1-yl) phenol (MMPP), a selective signal transducer and activator of transcription 3 (STAT3) inhibitor. We examined whether MMPP (5 mg/kg in drinking water for 1 month) prevents amyloidogenesis and cognitive impairment on AD model mice induced by intraperitoneal LPS (250 μg/kg daily 7 times) injections. Additionally, we investigated the anti-neuroinflammatory and anti-amyloidogenic effect of MMPP (1, 5, and 10 μg/mL) in LPS (1 μg/mL)-treated cultured astrocytes and microglial BV-2 cells. MMPP treatment reduced LPS-induced memory loss. This memory recovery effect was associated with the reduction of LPS-induced inflammatory proteins; cyclooxygenase-2 (COX-2) and inducible nitric oxide synthase (iNOS) as well as activation of microglial cells and astrocytes in the brain. Furthermore, MMPP reduced LPS-induced β-secretase and Aβ generation. In in vitro study, LPS-induced expression of inflammatory proteins and amyloidogenic proteins was decreased in microglial BV-2 cells and cultured astrocytes by MMPP treatment. Moreover, MMPP treatment suppressed DNA binding activities of the activation of STAT3 in in vivo and in vitro. These results indicated that MMPP inhibits LPS-induced amyloidogenesis and neuroinflammation via inhibition of STAT3.

**Electronic supplementary material:**

The online version of this article (doi:10.1007/s12017-017-8469-3) contains supplementary material, which is available to authorized users.

## Introduction

Alzheimer’s disease (AD) is a chronic neurodegenerative disease in people over 65 years old. Although there are many causes for AD, the main cause is the accumulation of beta-amyloid (Aβ) which is the most toxic to brain cells. Aβ peptides are generated from amyloid precursor protein (APP) by β-site APP-cleaving enzyme 1 (BACE1) and γ-secretase. BACE1 cleaves the APP, producing a soluble APP fragment (sAPPβ) and a carboxy-terminal 99 amino acid (C99) protein. C99 is then cleaved within the membrane by γ-secretase and generates Aβ and APP intracellular domain (AICD) (Gu et al. 2015; Holsinger et al. 2002). Additionally, Aβ molecules can form flexible soluble oligomers which may exist in several forms (Schnabel 2010). Misfolded oligomers cause cell death of brain (Pulawski et al. 2012).

Lipopolysaccharide (LPS), an endotoxin isolated from gram-negative bacteria, can induce neuronal damage and inflammatory responses (Qin et al. 2007). Our and other previous studies have proved that memory impairment and amyloidogenesis can be induced by systemic injections of LPS in in vivo and in vitro (Lee et al. 2008; Hauss-Wegrzyniak et al. 1998; DiCarlo et al. 2001; Gu et al. 2015). Other study also demonstrated that LPS administered into brain could induce amyloidogenesis and neuroinflammation (Biesmans et al. 2013; Choi et al. 2012; Noh et al. 2014; Chen et al. 2008). LPS induces accumulation of Aβs in both the cerebral cortex and hippocampus through increased β- and γ-secretase activities accompanied with the increased expression of APP, C99 and generation of Aβ as well as activation of astrocytes and microglia (Sheng et al. 2003; Pop et al. 2012; Henry et al. 2009). In a neurodegenerative disease, microglial cells and astrocytes are activated (Madeddu et al. 2015). LPS has been widely used to establish an experimental model of glial activation (Sola et al. 2002). LPS also augmented microglia activation in the central nervous system and accelerated the progression of a neurodegenerative disease (Masocha 2009; Qin et al. 2007). Activated astrocytes expressing glial fibrillary acidic protein (GFAP) are closely associated with AD pathology such as amyloid depositions and, in practice, increased amounts of GFAP were detected in the frontal cortex of AD patients (Korolainen et al. 2005; Cotter et al. 2001). During a neurological disease, members of the activation of signal transducer and activator of transcription (STAT) family of transcription factor have critical roles in inflammatory reactions through regulating many proteins and cytokines involved in neuroinflammation (Ban et al. 2014; Song et al. 2014). In the pathogenesis of AD, activation of the STAT3 signaling pathway has a critical role because STAT3 is associated with cytokine signaling during neuronal differentiation and inflammation (Wan et al. 2010). It was also reported that STAT3 is activated and elevated in hippocampal slices taken postmortem from AD patients and the brains of AD mouse models (Wan et al. 2010). STAT3-mediated transcriptional control of BACE1 has implications for AD pathogenesis through the elevation of Aβ generation (Wen et al. 2008). It is believed that interfering with the signaling network of STAT3 activation could be an effective approach in the treatment for AD by decreasing the level of Aβ gene expression (Song et al. 2014; Carret-Rebillat et al. 2015). It has been reported that several agents such as thiacremonone (Song et al. 2014; Yun et al. 2016), 2,4-bis(p-hydroxyphenyl)-2-butenal (Jin et al. 2013), ent-Sauchinone (Song et al. 2014), 4-O-Methylhonokiol (Lee et al. 2012b) decreased β-secretase activity through the inhibiting STAT3 activity in AD model.

In our previous study, we found that the significant anti-inflammatory properties of (E)-2, 4-bis(p-hydroxyphenyl)-2-butenal (BHPB) as inhibiting STAT3 activation in arthritis (Ban et al. 2014), cancer (Cho et al. 2014) and AD (Jin et al. 2013). Derivative of BHPB, 2,4-bis(4-hydroxyphenyl)but-2-enal diacetate (HPBD) also showed anti-neuroinflammatory and anti-amyloidogenic effects (Kim et al. 2014). However, these chemicals have fatal disadvantages such as instability of structure to use as a drug and weakened binding affinity to STAT3. The upgraded BHPB analogue, (E)-2-methoxy-4-(3-(4-methoxyphenyl) prop-1-en-1-yl) phenol (MMPP) is designed with modification of the conjugated α,β-unsaturated aldehyde moiety and protection of phenolic alcohols to various ethers. It can strongly bind to STAT3 through a direct molecular interaction with the hydroxyl residue in the core fragment (CF) of STAT3, so it can inhibit activation of STAT3. Furthermore, we carried out an *In silico* toxicology and ADME (absorption, distribution, metabolism, and extraction) evaluation previously by using computational ADME QSAR models, preADMET (http://preadmet.bmdrc.org) and StarDrop (http://www.optibrium.com/stardrop/stardrop-features.php), to predicted the ADME of MMPP (Son et al. 2016). Blood–Brain Barrier (BBB) penetration is represented as BB = [Brain]/[Blood], where [Brain] and [Blood] are the steady-state concentration of radiolabeled compounds in brain and peripheral blood. It was predicted that MMPP shows higher value with 0.92, which suggest that MMPP could penetrates easily to BBB (the value 1 means that 100% of compound can penetrate BBB). We therefore investigated whether MMPP suppresses LPS-triggered inflammatory responses and amyloidogenesis in both in vitro and in vivo.

## Materials and Methods

### Ethical Approval

The experimental protocols were carried out according to the guidelines for animal experiments of Institutional Animal Care and Use Committee (IACUC) of Laboratory Animal Research Center at Chungbuk National University, Korea (CBNUA-929-16-01). All efforts were made to minimize animal suffering, and to reduce the number of animals used. All mice were housed in three mice per cage with automatic temperature control (21–25 °C), relative humidity (45–65%), and 12 h light–dark cycle illuminating from 08:00 a.m. to 08:00 p.m. Food and water were available ad libitum. They were fed pellet diet consisting of crude protein 20.5%, crude fat 3.5%, crude fiber 8.0%, crude ash 8.0%, calcium 0.5%, phosphorus 0.5% per 100 g of the diet (collected from Daehan Biolink, Chungcheongbuk-do, Korea). During this study, all mice were specially observed for the normal body posture, piloerection, ataxia, urination, etc., 2 times per day.

## Materials

To synthesize MMPP, 4-Iodo-2-methoxyphenol (500 mg, 2.0 mmol, Sigma-Aldrich) and 4-allylanisole (296.4 mg, 2.0 mmol, Sigma-Aldrich) were added with triphenylphosphine (105 mg, 0.4 mmol), Pd(OAc)_2_ (44.9 mg, 0.2 mmol), and tributylamine (451 μL, 1.9 mmol) in a 25-mL round bottom flask and the reaction mixture was stirred for 2 h at 45 °C under argon atmosphere. The product was purified by flash silica gel chromatography using hexane and ethyl acetate (3:1 mixture v/v) as the mobile phase. Reduction of the alkene or the aldehyde of α, β -unsaturated aldehyde moiety resulted in stable compounds as protecting the phenolic alcohol from ether.

### Animal Experiments

Eight-to-ten week-old-male imprinting control region (ICR) mice (Hajinbiotech, Gyeonggi-do, Korea) were maintained and handled in accordance with the humane animal care and use guidelines of Korean FDA. To induce neuroinflammatory cognitive impairment model, LPS (250 μg/kg) was administered intraperitoneally (Lee et al. 2013a). Previous studies revealed that LPS injection caused amyloidogenesis and memory loss in mice brain through NF-κB activation (Lee et al. 2012a; Choi et al. 2012; Lee et al. 2013b; Fan et al. 2014). Also, STATs have been involved in amyloidogenesis through the modulation of secretases (Hashimoto et al. 2005). There are three groups: (I) Control group, (II) LPS group, and (III) MMPP + LPS group where each group was assigned 10 mice. The MMPP was given to (III) group in drinking water daily at a dose of 5 mg/kg for 4 weeks. Intraperitoneal (i.p.) injection of LPS (250 μg/kg) was administered except for the control group on the 4th week for 7 days. The doses of MMPP are referred to in a previous study (Jin et al. 2013). The behavioral tests of learning and memory capacity were then assessed using three tests (water maze, probe and passive avoidance test). The water maze test was performed even days after the MMPP and LPS administration. The probe test was performed one day after the water maze test. The passive avoidance test was performed one day after the probe test.

### Morris Water Maze

The water maze test is also a commonly accepted method for memory test, and we performed this test as described by Morris et al. (Morris 1984). Maze testing was fulfilled by the SMART-CS (Panlab, Barcelona, Spain) program and equipment. A circular plastic pool (height: 35 cm, diameter: 100 cm) was filled with squid ink water kept at 22–25 °C. An escape platform (height: 14.5 cm, diameter: 4.5 cm) was submerged 1–1.5 cm below the surface of the water in position. On training trials, the mice were placed in a pool of water and allowed to remain on the platform for 120 s and were then returned to the cage. The mice that did not find the platform within 60 s were placed on the platform for 10 s at the end of trial. The mice that did find the platform and stayed on it for 3 s within the 60 s were placed on the platform for seven more seconds at the end of trial. These trials were performed on a single platform and at two rotational starting positions. Escape latency and escape distance of each mouse were monitored by a camera above the center of the pool connected to a SMART-LD program (Panlab, Barcelona, Spain).

### Probe Test

To assess memory retention, a probe test was performed 24 h after the water maze test. The platform was removed from the pool which was used in the water maze test, and the mice were allowed to swim freely. The swimming pattern of each mouse was monitored and recorded for 60 s using the SMART-LD program (Panlab). Retained spatial memory was estimated by the time spent in the target quadrant area.

### Passive Avoidance Performance Test

The passive avoidance test is generally accepted as a simple method for testing memory. The passive avoidance response was determined using a “step-through” apparatus (Med Associates Inc, Vermont, USA) that is divided into an illuminated compartment and a dark compartment (each 20.3 × 15.9 × 21.3 cm) adjoining each other through a small gate with a grid floor, 3.175 mm stainless steel rods set 8 mm apart. On the first day, the mice were placed in the illuminated compartment facing away from the dark compartment for the training trial. When the mice moved completely into the dark compartment, it received an electric shock (0.45 mA, 3 s duration). Then, the mice were returned to their cage. One day after training trial, the mice were placed in the illuminated compartment and the latency period to enter the dark compartment defined as “retention” was measured. The time when the mice entered into the dark compartment was recorded and described as step-through latency. The retention trials were set at a cutoff time limit of 3 min.

### Brain Collection and Preservation

After behavioral tests, mice were perfused with phosphate-buffered saline (PBS, pH 7.4) with heparin under inhaled CO_2_ anesthetization. The brains were immediately removed from the skulls and divided into left brain and right brain. One stored at −80 °C, the other were fixed in 4% paraformaldehyde for 72 h at 4 °C and transferred to 30% sucrose solutions.

### Astrocytes and Microglial BV-2 Cells Culture

Astrocytes were prepared from the cerebral cortex of rat embryos (E18). After the skull was cut and the skin was opened, the brain was released from the skull cavity. After washing with PBS, the cerebrum was separated from the cerebellum and brain stem, and the cerebral hemispheres were separated from each other by gently teasing along the midline fissure with the sharp edge of forceps. The meninges were gently peeled from the individual cortical lobes and the cortices were dissociated by mechanical digestion [using the cell strainer (BD Bioscience, Franklin Lakes, NJ, USA)]. The resulting cells were centrifuged (1500 rpm, 5 min), resuspended in serum-supplemented culture media, and plated into 100 mm dishes. The cells were seeded on culture flasks T-75 and incubated in Dulbecco’s modified eagle medium (DMEM)/F-12 (Invitrogen, Carlsbad, CA) containing 10% fetal bovine serum (FBS) (Invitrogen). The culture medium was replaced every 3 days thereafter. After 14 days, the cultures became confluent and loosely attached microglia and oligodendrocyte precursor cells were removed from the cell monolayer using shaking incubator (37 °C, 350 RPM, 2–4 h). Astrocytes were subsequently detached using trypsin–EDTA and plated into 100 mm cell culture dishes. The percentage of astrocytes in our culture system is more than 95%. Microglial BV-2 cells were maintained with serum-supplemented culture media of DMEM supplemented with FBS (10%) and antibiotics (100 units/mL). The microglial BV-2 were incubated in the culture medium in a humidified incubator at 37 °C and 5% CO_2_. The cultured cells were treated with several concentrations (1.0, 5.0 10.0 μg/mL) of MMPP, 2 h before LPS (1 μg/mL) addition, and astrocytes were treated in a basal medium that contains 1% FBS. The cells were harvested after 24 h.

### Immunohistochemical Staining

After being transferred to 30% sucrose solutions, brains were cut into 20 μm sections by using a cryostat microtome (Leica CM 1850; Leica Microsystems, Seoul, Korea). After two washes in PBS (pH 7.4) for 10 min each, endogenous peroxidase activity was quenched by incubating the samples in 3% hydrogen peroxide in PBS for 20 min, and then two washes in PBS for 10 min each. The brain sections were blocked for 1 h in 5% bovine serum albumin (BSA) solution, and incubated overnight at 4 °C with a mouse polyclonal antibody against GFAP (1:300; Santa Cruz Biotechnology, Inc., Santa Cruz, CA, USA), inducible nitric oxide synthase (iNOS) (1:300; Novus Biologicals, Inc., Littleton), a rabbit polyclonal antibody against cyclooxygenase-2 (COX-2) (1:300; Cell Signaling Technology, Inc., Beverly, MA, USA), and a goat polyclonal antibody against ionize calcium-binding adapter molecule 1 (Iba-1) (1:300; Abcam, Inc., Cambridge, MA, USA). After incubation with the primary antibodies, brain sections were washed twice in PBS for 10 min each. After washing, brain sections were incubated for 1–2 h at room temperature with the biotinylated goat anti-rabbit or goat anti-mouse or donkey anti-goat IgG-horseradish peroxidase (HRP) secondary antibodies (1:500; Santa Cruz Biotechnology, Inc., Santa Cruz, CA, USA). Brain sections were washed thrice in PBS for 10 min each, and visualized by a chromogen DAB (Vector Laboratories) reaction for up to 10 min. Finally, brain sections were dehydrated in ethanol, cleared in xylene, mounted with Permount (Fisher Scientific, Hampton, NH), and evaluated on a light microscopy (Microscope Axio Imager.A2, Carl Zeiss, Oberkochen, Germany) (× 50 and × 200). We investigated the region of brain from CA3 and DG region in hippocampus for anatomical studies. CA3 and DG network is the most critical for contributing to memory storage and retrieval of memory sequences.

### Fluorescence Microscopy

The fixed cells and brain sections were exposed to the following primary antibodies: GFAP (1:300, Santa Cruz Biotechnology Inc. Santa Cruz, CA, USA), Iba-1 (1:300, Abcam, Inc., Cambridge, MA, USA), and Aβ_1–42_ (1:300, Abcam ab10148, Inc., Cambridge, MA, USA) at room temperature for 2 h. The Aβ_1–42_ antibody is reactive with Aβ_1–42_ and does not cross-react with Aβ_1–42_, full-length APP, sAPP beta or sAPP alpha. After incubation, the cells were washed twice with ice-cold PBS and incubated with an anti-rabbit or mouse or goat secondary antibody conjugated to Alexa Fluor 488 or 568 nm (Invitrogen-Molecular Probes, Carlsbad, CA) at room temperature for 1 h. Immunofluorescence images were acquired using an inverted fluorescent microscope Zeiss Axiovert 200 M (Carl Zeiss, Thornwood, NY) (× 200). We investigated the region of brain from CA3 and DG region in hippocampus for anatomical studies. CA3 and DG network is the most critical for contributing to memory storage and retrieval of memory sequences.

### Thioflavin S Staining

After being transferred to 30% sucrose solutions, brains were cut into 20 μm sections by using a cryostat microtome (Leica CM 1850; Leica Microsystems, Seoul, Korea). After washes in distilled water for 5 min, brain sections were transferred to gelatin-coated slide and placed in 1% thioflavin S (Thioflavin S, Sigma, St Louis, MO, USA) for 5 min. Brain sections were then washed in distilled water and then dehydrated through ascending grades of ethanol, 50, 70, 90, and 100% ethanol for 2 min in each grade. The sections were then mounted in a mounting medium (Fluoromount™ Aqueous Mounting Medium, Sigma, St Louis, MO, USA). The thioflavin S staining was examined using a fluorescence microscope (Axio Observer A1, Carl Zeiss, Oberkochen, Germany) (× 100). We investigated the region of brain from CA3 and DG region in hippocampus for anatomical studies. CA3 and DG network is the most critical for contributing to memory storage and retrieval of memory sequences.

### Measurement of Aβ_1–42_

Lysates of brain tissue were obtained through protein extraction buffer containing protease inhibitor. Aβ_1–42_ levels were determined using each specific mouse amyloid-beta peptide 1-42 ELISA Kit (CUSABIO, CSB-E10787 m). Protein was extracted from brain tissues using a protein extraction buffer (PRO-PREPTM, Intron Biotechnology, Korea), incubated on ice for 1 h and centrifuged at 13,000 × g for 15 min at 4 °C. In brief, 100 μL of sample was added into a precoated plate and incubated for 2 h at 37 °C. After removing any unbound substances, a biotin-conjugated antibody specific for Aβ_1–42_ was added to the wells. After washing, avidin-conjugated HRP was added to the wells. Following a wash to remove any unbound avidin-enzyme reagent, a substrate solution was added to the wells and color developed in proportion to the amount of Aβ_1–42_ bound in the initial step. The color development was stopped and the intensity of the color was measured.

### Assay of β-secretase Activities

β-secretase activity in the mice brains was determined using a commercially available β-secretase activity kit (Abcam, Inc, Cambridge, MA, USA). Solubilized membranes were extracted from brain tissues using β-secretase extraction buffer, incubated on ice for 1 h and centrifuged at 5000 × g for 10 min at 4 °C. The supernatant was collected. A total of 50 μL of sample (total protein 100 μg) or blank (β-secretase extraction buffer 50 μL) was added to each well (used 96-well plate) followed by 50 μL of 2  ×  reaction buffer and 2 μL of β-secretase substrate incubated in the dark at 37 °C for 1 h. Fluorescence was read at excitation and emission wavelengths of 335 and 495 nm, respectively, using a fluorescence spectrometer (Gemini EM, Molecular Devices, California, USA).

### Nuclear Extraction and Gel Mobility Shift Assay

Gel mobility shift assay was conducted using a slight modification of a previously described method (Lee et al. 2011). In brief, 10 μg of nuclear protein of astrocytes, microglial BV-2 cells and brain tissues was incubated in 25 μL of total volume of incubation buffer (10 mmol/L Tris, pH 7.5, 100 mmol/L NaCl, 1 mmol/L dithiothreitol, 4% glycerol, 80 mg/L salmon sperm DNA) at 4 °C for 15 min followed by another 20 min incubation with 9.25 mBq [-^32^P] ATP-labeled oligonucleotide containing STAT3 binding site at room temperature. The DNA–protein binding complex was electrophoretically resolved on a 5% non-denatured polyacrylamide gel at 150 volts for 90 min. The gels were dried and autoradiographed using Kodak MR film at −80 °C overnight.

### Western Blotting

In in vivo and in vitro study, for comparing the expression of protein levels through Western blotting, we selected and used 3 of 10 mice brain from each group. An equal amount of total protein (20 μg) was resolved on 8–15% sodium dodecyl sulfate polyacrylamide gel and then transferred to a nitrocellulose membrane (Hybond ECL; Amersham Pharmacia Biotech, Piscataway, NJ, USA). The membranes were blocked for 1 h in 5% skim milk solution, and incubated overnight at 4 °C with specific antibodies. To detect target proteins, specific antibodies against C99 (1:1000, EMD Millipore, Billerica, MA, USA), APP, iNOS (1:1000, Novus Biologicals, Inc., Littleton), BACE1, Iba-1 (1:1000, Abcam, Inc., Cambridge, MA, USA), COX-2 (1:1000, Cell Signaling Technology, Inc., Beverly, MA, USA), GFAP, STAT3, p-STAT3, β-actin (1:1000, Santa Cruz Biotechnology Inc. Santa Cruz, CA, USA) were used. The blots were then incubated with the corresponding conjugated goat anti-rabbit or goat anti-mouse or donkey anti-goat IgG-HRP (1:5000; Santa Cruz Biotechnology Inc. Santa Cruz, CA, USA) secondary antibodies. Immunoreactive proteins were detected with an enhanced chemiluminescence Western blotting detection system. The relative density of the protein bands was scanned and quantified by ImageJ (Wayne Rasband, National Institutes of Health, Bethesda, MD) was used.

### Nitrite Assay

Astrocytes and microglial BV-2 cells were plated at a density of 5 × 10^5^ cells/well in 6-well plates per 2 mL medium for 24 h. After removing the culture medium, the cells were then treated with LPS (1 μg/mL) and MMPP (1, 5, 10 μg/mL) per 2 mL medium for 24 h. The nitrite in the supernatant was assessed using a NO detection kit (iNtRON Biotechnology, Seongnam, Korea), according to the manufacturer’s instructions. Finally, the resulting color was assayed at 520 nm using a microplate absorbance reader (VersaMax ELISA, Molecular Devices, California, USA).

### RNA Isolation and Quantitative Real-Time RT-PCR

Cell RNA was isolated from homogenized microglial BV-2 cells and primary cell cultured astrocytes using RiboEX (Gene All, Seoul, Korea), and total RNA (0.2 μg) was reverse-transcribed into cDNA according to the manufacturer’s instructions using Applied Biosystems (Foster City, CA, USA). For the quantitative, real-time, reverse transcriptase polymerase chain reaction (PCR) assays, the linearity of the amplifications of IL-6, IL-10, and β-actin cDNAs was established in preliminary experiments. All signal mRNAs were normalized to β-actin mRNA. cDNAs were amplified by real-time PCR in duplicate with QuantiNova SYBR green PCR kit (Qiagen, Valencia, CA, USA). Each sample was run with the following primer sets: IL-6, 5′-GAGGATACCACTCCCAACAGACC-3′ (sense), 5′-AAGTGCATCATCGTTGTTCATACA-3′ (antisense); IL-10, 5′-TCTGAGCCACTCACATCTGC-3′ (sense), 5′-TCAGGGGAACTGCTAGTGCT-3′ (antisense); β-actin: 5′-TGGAATCCTGTGGCATCCATGAAAC-3′ (sense), 5′-TAAAACGCAGCTCAGTAACAGTCCG-3′ (antisense).

### Plasmid Construction

The region of *Mus musculus* STAT3 was amplified by PCR using full-length *Mus musculus* STAT3 cDNA as a template. The purified PCR products were double-digested with EcoRI and XhoI and then subcloned into the pcDNA3.1 vector. The pcDNA3.1 plasmid contains a cytomegalovirus promoter, pUC origin, and ampicillin-resistance gene. STAT3 (T456A) mutagenesis was generated by Cosmogenetech Co. (Seoul, Korea) and the mutant was systematically checked by sequencing. RAW264.7 cells were plated at a density of 1 × 10^5^ cells per 24-well plate. After 24 h of growth to 90% confluence, the cells were transfected with mutant STAT3 plasmid (T456A; T, a core amino acid of DNA binding domain was replaced with un related amino acid A) using Lipofectamine LTX & PLUS (Invitrogen Co., Carlsbad, CA, USA) in OPTI-MEM media (Invitrogen) according to the manufacturer’s instructions.

### Statistical Analysis

For the measurement of the image data, ImageJ (Wayne Rasband, National Institutes of Health, Bethesda, MD) was used. Group differences were analyzed by one-way ANOVA followed by bonferroni’s post hoc analysis using GraphPad Prism 5 software (Version 5.02, GraphPad software, Inc., La Jolla, USA).

## Results

### MMPP Treatment Inhibits Memory Impairment in LPS-Treated Mice

**Fig. 1:**
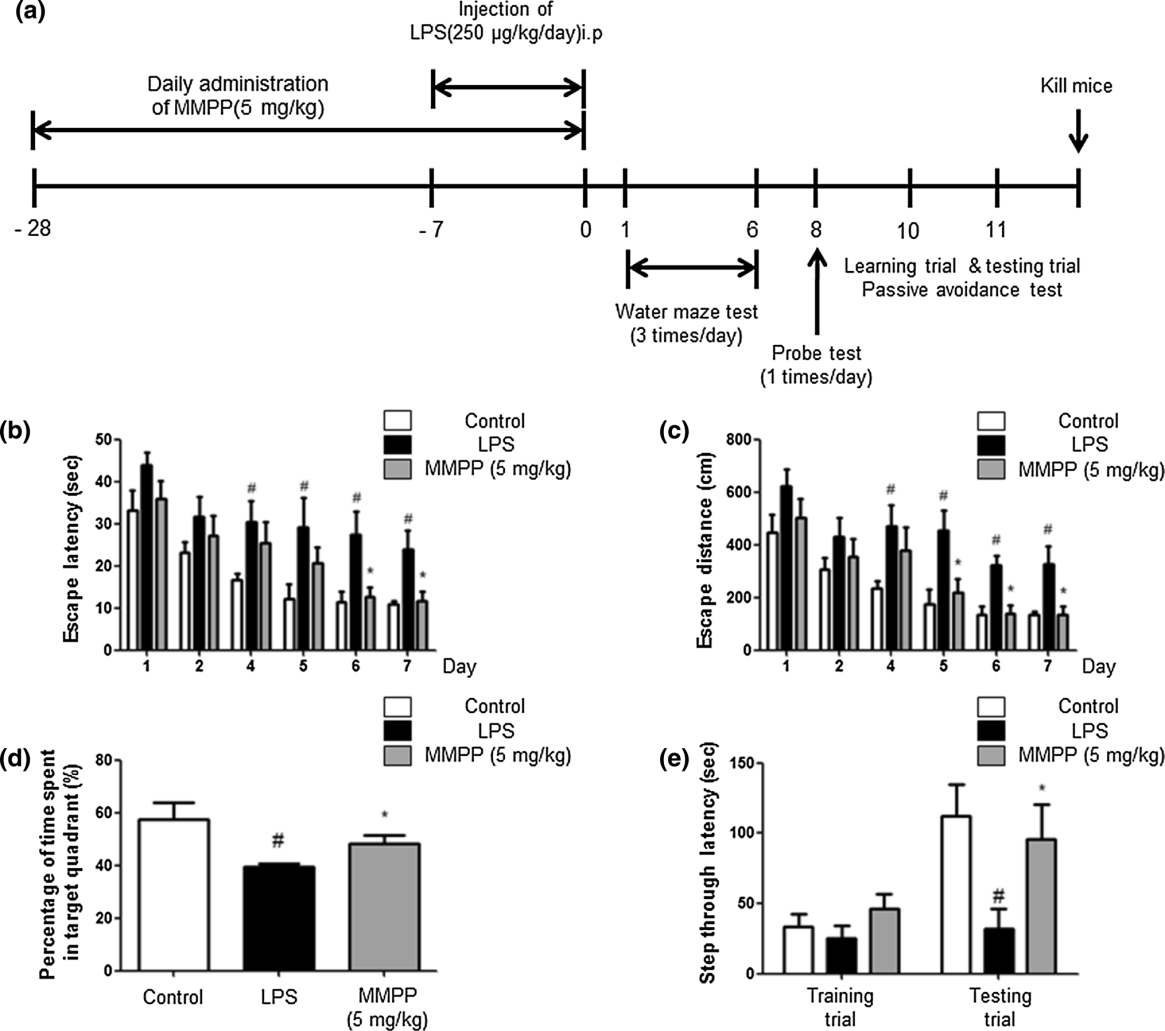
Effect of MMPP on improvement of memory impairment in LPS-injected mice. (**a**) Timeline depicts the treatment of MMPP and assessments of cognitive functions of mice. The mice (*n* = 10) were treated MMPP by drinking water daily at dose of 5 mg/kg for 4 weeks. I.p. injection of LPS (250 μg/kg) was administered except for control group on the 4th week for 7 days, and memory tests were conducted. The training trial was performed three times a day for 7 days. Swimming time (**b**) and swimming distance (**c**) to the platform were automatically recorded. The time spent in the target quadrant and target site crossing within 60 s was represented (**d**). To perform the passive avoidance test, step-through method was used (**e**). Each value is presented as mean ± S.D. from ten mice. ^#^Significantly different to control mice (*P* < 0.05), *Significantly different to LPS-injected mice (*P* < 0.05)

The cognitive impairment and the effect of memory improvement by MMPP were estimated by using the water maze and passive avoidance performance. We investigated the ability of mice to learn and recall spatial memory through escape latency and distance in the water maze. The LPS-injected mice (23.94 ± 4.40) learned more slowly than control mice (10.90 ± 0.86) and MMPP-treated mice (11.79 ± 2.101) (*F* = 5.01, *p* value = 0.03) exhibited a reduction in escape latency over the training period (Fig. 1b). MMPP-treated mice (136.6 ± 28.32) also showed a shorter escape distance (Fig. 1c) compared to LPS-injected mice (325.3 ± 68.17) (*F* = 6.62, *p* value = 0.03). After the final day of the water maze, we performed a probe test to calculate the time spent in the target quadrant zone for testing maintenance of memory. MMPP-treated mice (48.28 ± 3.06) spent much more time in the quadrant zone than LPS-injected mice (39.44 ± 1.20) (Fig. 1d). Then, we tested the mice to evaluate how long they can remember through the passive avoidance test. Although there was no significant difference on the learning trial, MMPP-treated mice (95.60 ± 24.81) recorded increased step-through latency compared with the LPS-treated group (32.09 ± 13.93) on the testing trial (Fig. 1e).

### MMPP Treatment Inhibits Aβ Accumulation and Amyloidogenesis in Brains of LPS-Treated Mice

**Fig. 2:**
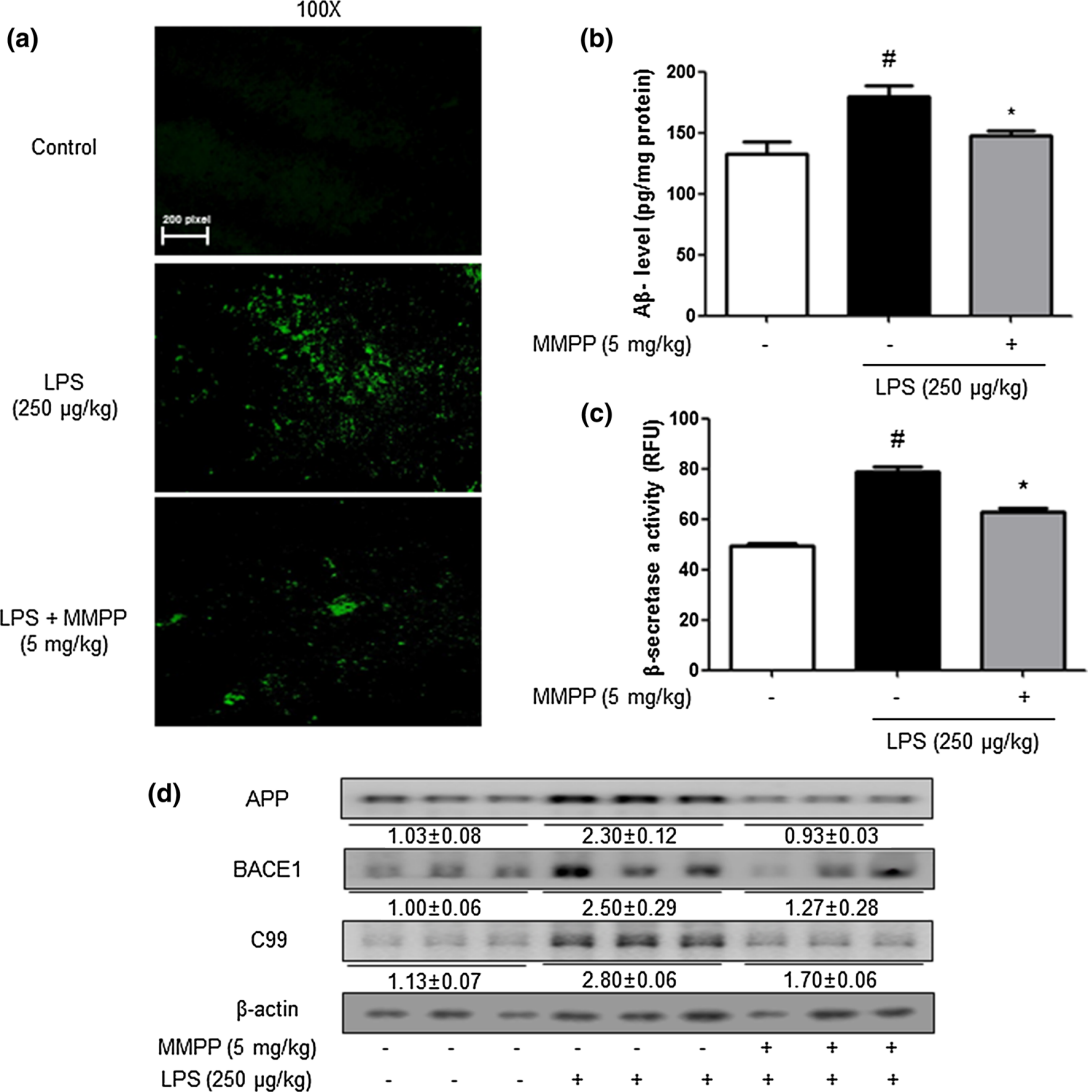
Inhibitory effects of MMPP on accumulation of Aβ_1-42_ in the brain of LPS-injected mice. (**a**) Aβ accumulation in the brains of LPS-injected mice was determined by thioflavin S staining. The levels of Aβ_1-42_ in mice brain (*n* = 5) were measured by ELISA (**b**). The activity of β-secretase in mice brain (*n* = 5) was investigated using assay kit (**c**). The expression of APP, BACE1 and C99 was detected by Western blotting using specific antibodies in the mouse brain (**d**). For the cropped images, samples were run in the same gels under same experimental conditions and processed in parallel. Each blot is representative for three experiments

We detected how much Aβs accumulated in the brain through thioflavin S staining that is dyed with beta sheet-rich structures of Aβ. We investigated the region of brain from CA3 and DG region in hippocampus for anatomical studies. CA3 network is the most critical for encoding novel information quickly into the hippocampal memory, so it contributes to memory storage and retrieval of memory sequences (Kesner 2007; Cherubini and Miles 2015). DG performs translating neural codes for memory formation (Madronal et al. 2016). Furthermore, it was reported that DG inhibition is a loss of learning-associated plasticity (Lisman 1999). As inspecting these regions of hippocampus, elucidation of MMPP on social recognition deficits will help pave the road for future treatment evaluation and drug development. Compared to the non-treated mice brain, much higher accumulation of Aβs was found in the brains of LPS-treated mice compared to non-treated mice brains. However, lower accumulation of Aβs was detected in the brains of MMPP-treated mice (Fig. 2a). To investigate whether Aβ deposition by immunohistochemical analysis was paralleled with Aβ level in the brain, quantitative analyses of the level of Aβ was performed using ELISA. Aβ level in the brains of LPS-injected mice were significantly higher compared to the level of Aβ in the non-treated mice brains, but it was reduced in the MMPP-treated mice brains (Fig. 2b). Since Aβs are produced by activated β-secretases, we measured the activity of β-secretase in the hippocampus. The activity of β-secretase was increased in the brains of LPS-injected mice, while the activity was decreased in MMPP-treated mice brains significantly (Fig. 2c). To clarify whether MMPP could influence inhibition of amyloidogenesis in the brain, we performed a Western blot assay. LPS-elevated expression of APP, BACE1 and C99 was significantly decreased by MMPP treatment (Fig. 2d).

**Fig. 3:**
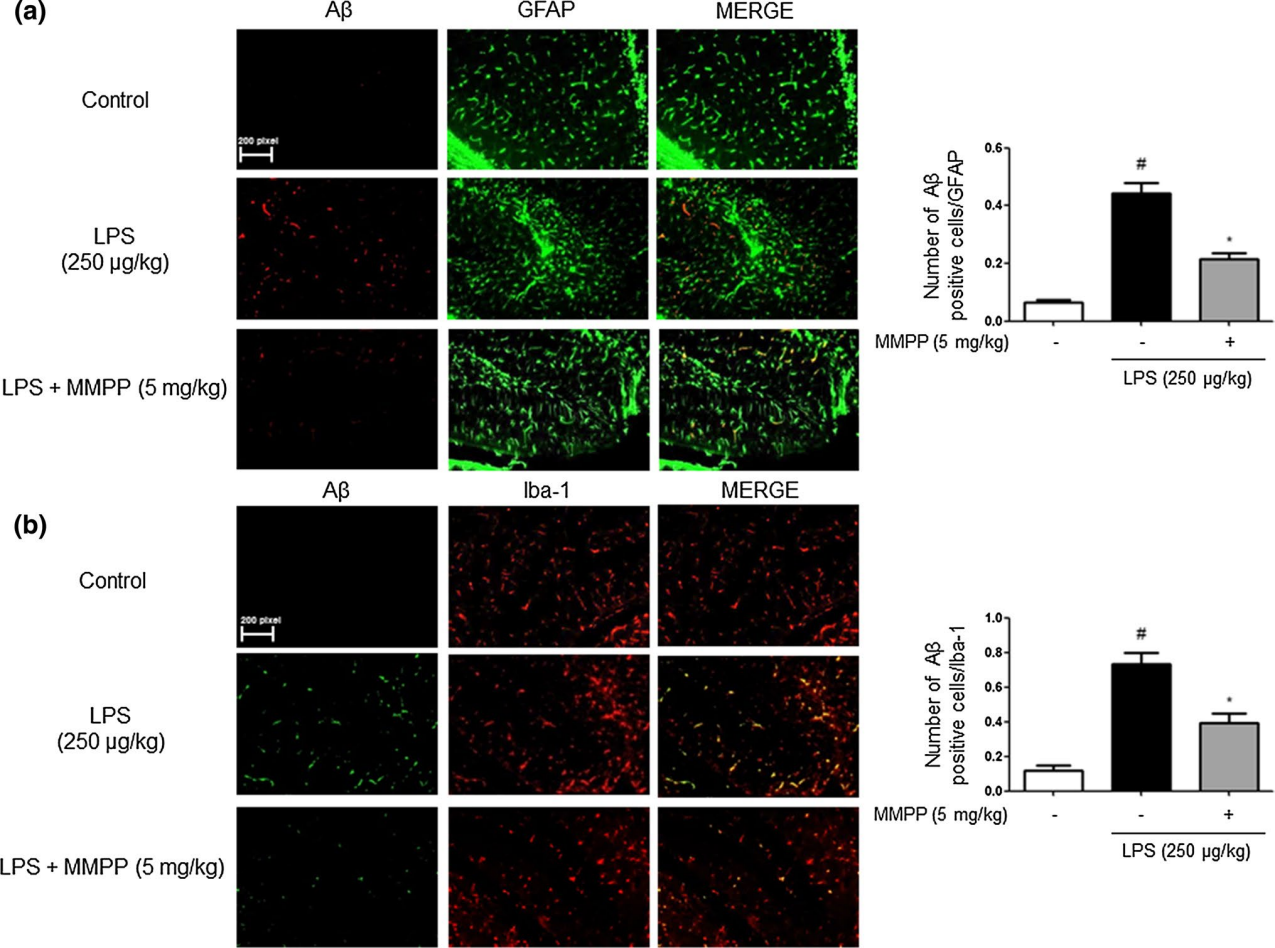
Inhibitory effect of MMPP on LPS-induced expression of Aβ_1-42_ in both GFAP and Iba-1-positive mice brain. Staining was performed in 20-μm-thick sections of mice brain. Confocal microscope observation was performed as described in the Methods section. Immunostaining of GFAP (green) and Aβ_1−42_ (red) protein in the hippocampus was performed with specific primary antibodies, and fluorescence was developed using Alexa 488-conjugated anti-goat and Alexa 568-conjugated anti-rabbit secondary antibodies (**a**). Iba-1 (red) and Aβ_1−42_ (green) protein in the hippocampus was performed with specific primary antibodies, and fluorescence was developed using Alexa 488-conjugated anti-mouse and Alexa 568-conjugated anti-rabbit secondary antibodies (**b**). Similar patterns were observed in five mice brain

We investigated whether the numbers of activated (GFAP-positive) astrocytes and accumulation of Aβ (Aβ-positive cells) were concomitantly increased by LPS, and whether this activation could be reduced by MMPP, thereby reducing Aβ-level. GFAP and Aβ double immunofluorescence method were used for the detection of immunoreactive cells. We investigated CA3 and DG region in hippocampus of brain for anatomical studies. The co-reactive cell number for both markers was significantly increased by LPS injection, but was decreased by MMPP treatment (Fig. 3a). We also investigated the inhibitory effects of MMPP on the activation of microglial cells. The co-reactive cell number for both activation of microglia (Iba1-postive cells) and Aβ accumulation (Aβ-positive cells) was also increased by LPS compared to the number in the non-treated mice brains, but was decreased by MMPP treatment (Fig. 3b).

### MMPP Treatment Downregulates STAT3 Activation in LPS-Treated Mice Brain

**Fig. 4:**
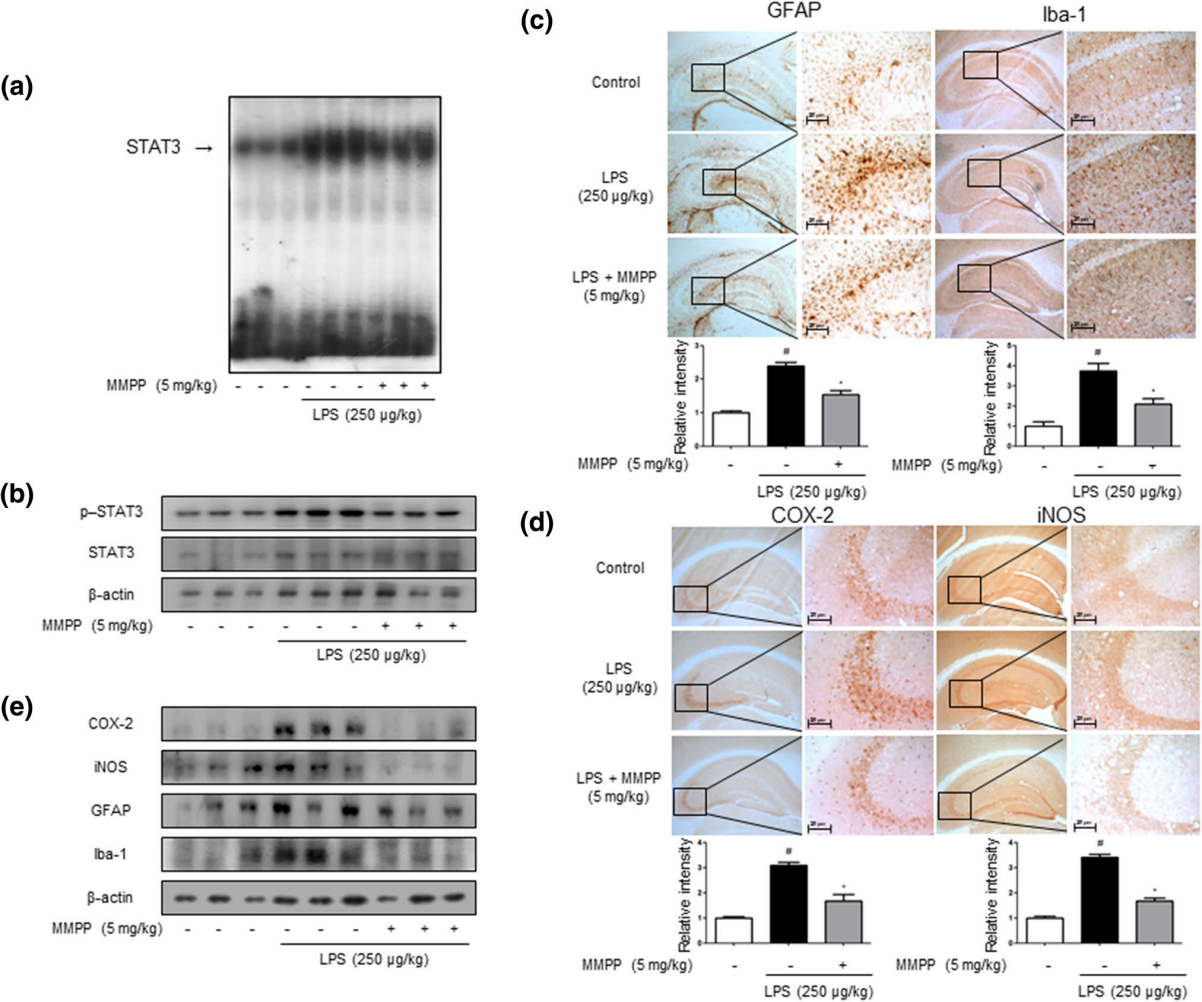
Inhibitory effect of MMPP on STAT3 translocation related DNA binding activity and anti-inflammatory effect in LPS-injected mouse brain. Effect of MMPP on STAT3 activity in the LPS-injected mouse brain was determined by gel electromobility shift assay (EMSA) (**a**). Representative results were obtained from at least three different sets of experiment (*n* = 3). The MMPP-treated mouse brain extracts were prepared, and phospho-STAT3 and STAT3 level were detected by Western blot (**b**). Each blot was representative of three experiments. Immunostaining of GFAP, Iba-1, COX-2, and iNOS proteins in the hippocampus was performed in 20-μm-thick sections of mice brain with specific primary antibodies and the biotinylated secondary antibodies (**c**, **d**). The expression of COX-2, iNOS, GFAP and Iba-1 was detected by Western blotting using specific antibodies in the mice brain. Each blot is representative of three experiments (**e**). For the cropped images, samples were run in the same gels under same experimental conditions and processed in parallel. Each band is representative for three experiments

We studied whether MMPP inhibits STAT3 which is an important contributor in inflammatory responses as well as amyloidogenesis. We investigated the effects of MMPP on STAT3 activation in LPS-treated mice. The DNA binding activity of STAT3 was increased by LPS treatment, but the DNA binding activity of STAT3 was inhibited by MMPP treatment (Fig. 4a). Western blot analysis revealed that p-STAT3 level was also significantly decreased in the brains of MMPP-treated mice compared with LPS-injected mice brains (Fig. 4b).

### MMPP Reduces Neuroinflammation in Brain of LPS-Treated Mice

To investigate the activation of astrocytes and microglia cells as well as neuroinflammation, we performed immunohistochemistry (IHC) and Western blotting to detect the expression of GFAP (a marker of astrocyte activation), Iba-1 (a marker of microglia cell activation) and inflammatory proteins (iNOS and COX-2) in the brain. The GFAP-reactive cell number and Iba-1-reactive cell number were reduced in the mice brains with the treatment of MMPP compared to those in LPS-injected mice which showed much higher numbers of cells reactive for these marker proteins compared to non-treated mice brains (Fig. 4c). The expression of GFAP and Iba-1 was also significantly decreased in the brains of MMPP-treated mice than LPS-injected mice brains (Fig. 4d). We also investigated the inhibitory effect of MMPP in neuroinflammation; the expression of iNOS and COX-2 were determined. Treatment of LPS elevated the expression of inflammatory proteins (iNOS and COX-2), GFAP and Iba-1, but the expressions were significantly reduced by the treatment of MMPP (Fig. 4e).

### MMPP Inhibits Amlyloidogenesis and Neuroinflammation in Cultured Astrocytes and Microglial Cells

**Fig. 5:**
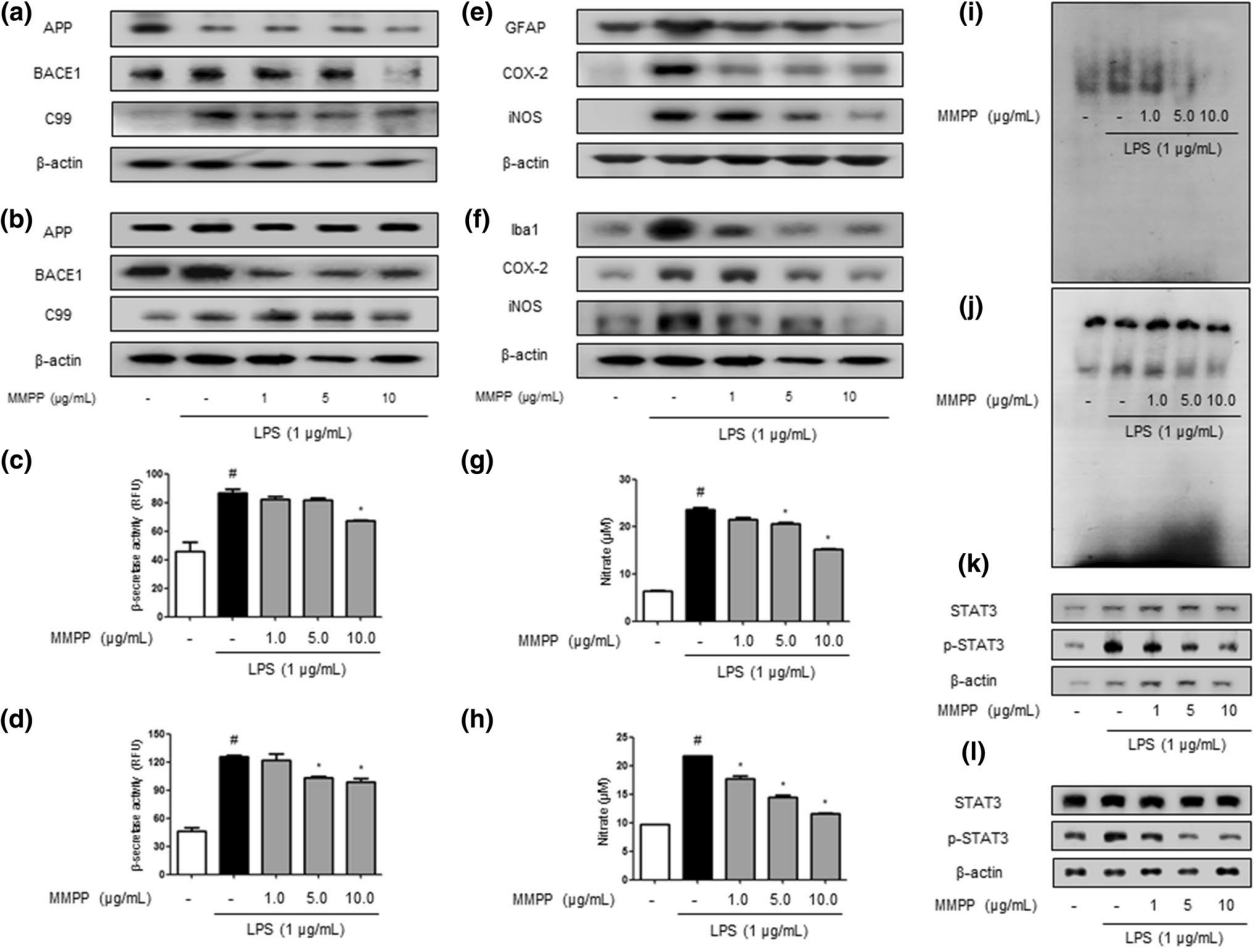
Inhibitory effect of MMPP on amyloidogenesis and STAT3 translocation in astrocytes and microglia cells. The expression of APP, BACE1 and C99 was detected by Western blotting using specific antibodies in astrocytes (**a**) and microglia cells (**b**). Each blot is representative of three experiments. The activity of β-secretase was investigated using assay kit in astrocytes (**c**) and microglia cells (**d**). Values are presented as mean ± S.D. of the three independent experiments performed in triplicate. ^#^ *p* < 0.05 compared to control, *p < 0.05 compared to LPS. Iba-1, COX-2, and iNOS proteins were detected by Western blotting using specific antibodies in astrocytes (**e**) and microglia cells (**f**). NO level was measured in astrocytes (**g**) and microglia cells (**h**). Activation of STAT3 was investigated using EMSA in astrocytes (**i**) microglial cells (**j**) were determined and the expression of STAT3 and phopho-STAT3 was also detected by Western blotting using specific antibodies (**k**), (**l**). For the cropped images, samples were run in the same gels under same experimental conditions and processed in parallel. Each band is representative for three experiments

The inhibitory effects of MMPP on amyloidogenesis and neuroinflammation in vitro were also investigated. To find anti-amyloidogenesis of MMPP in primary cultured astrocytes and microglial BV-2 cells, both cells were treated with 1 μg/mL of LPS and 1, 5 and 10 μg/mL of MMPP. The levels of BACE1 and C99 protein were increased in LPS-treated cells, whereas the expressions were reduced by MMPP treatment. However, the expression of APP was not significantly changed in astrocytes (Fig. 5a) and microglial BV-2 cells (Fig. 5b). MMPP also decreased LPS-induced β-secretase activity in MMPP-treated astrocytes (Fig. 5c) and BV-2 cells (Fig. 5d) in a concentration-dependent manner.

**Fig. 6:**
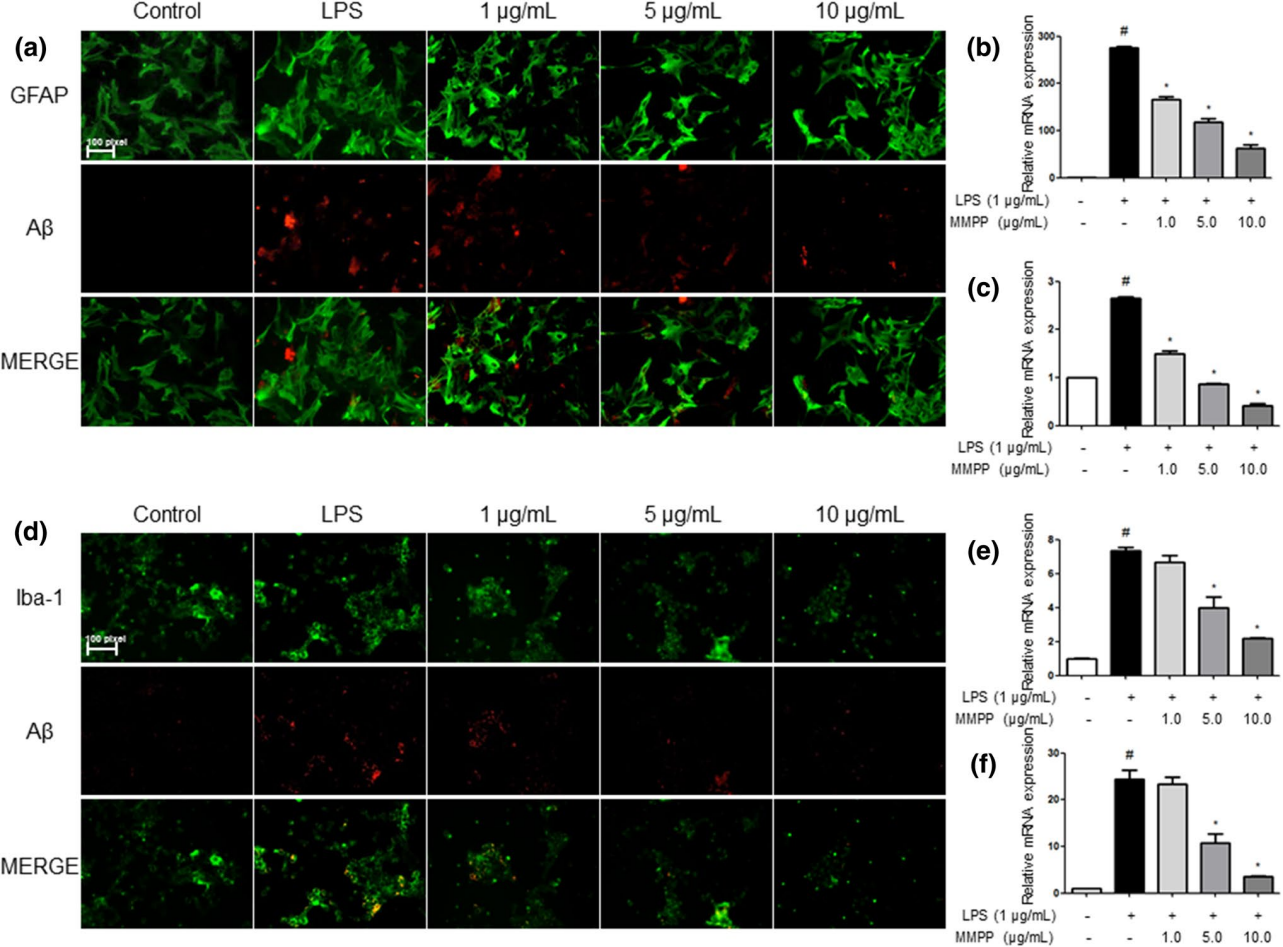
Inhibitory effect of MMPP on amyloidogenesis and neuroinflammatory responses in astrocytes and microglia cells. The cultured astrocytes were incubated with anti-GFAP (green) and anti- Aβ_1−42_ (red) primary antibodies (**a**), and microglial BV-2 cells were incubated with anti-Iba-1 (green) and anti- Aβ_1−42_ (red) primary antibodies (**d**). Fluorescence was developed using Alexa 488-conjugated anti-mouse and goat and Alexa 568-conjugated anti-rabbit secondary antibodies. mRNA levels of IL-6 (**b**) and IL-10 (**c**) were detected by qRT-PCR in primary cultured astrocytes. mRNA levels of IL-6 (**e**) and IL-10 (**f**) were detected by qRT-PCR in microglial-BV2 cells. Further, ^#^ *P* < 0.05 versus control, and *P < 0.05 versus LPS-treated control as determined by one-way ANOVA

Furthermore, it was detected that the nitrate level was decreased dose dependently in astrocytes (Fig. 5g), and microglial BV-2 cells (Fig. 5h). Then, we detected the expression of inflammatory proteins (iNOS, COX-2) as well as marker proteins of astrocytes (GFAP) and microglia (Iba-1) by Western blotting. The MMPP reduced LPS-induced increased expression of inflammatory proteins in a dose-dependent manner in astrocytes (Fig. 5e) and microglial BV-2 cells (Fig. 5f). We also discovered that MMPP inhibited LPS-induced STAT3 activity and the translocation of STAT3 protein to the nucleus in both astrocytes (Fig. 5i, k) and microglial BV-2 cells (Fig. 5j, l) through Western blot and EMSA. We detected double immunofluorescence in both the primary cultured astrocytes and microglia cells by confocal microscope analysis. Co-expression of GFAP (astrocytes marker) and Aβ was increased by LPS, which was decreased by MMPP treatment in astrocytes (Fig. 6a). Co-expression of Iba-1 (microglia cell marker) and Aβ in microglial BV-2 cells was also reduced by treatment of MMPP (Fig. 6d). We investigated expression levels of pro- and anti-inflammatory cytokines-related factors IL-6 and IL-10 in astrocytes and microglial BV-2 cells following MMPP treatment. Our results suggested that MMPP treatment decreased LPS-induced mRNA levels of IL-6 (Fig. 6b, e) and IL-10 (Fig. 6c, f) in a dose dependently in both primary cultured astrocytes and microglial-BV2 cells.

### MMPP Suppresses STAT3 Signaling by Interacting with STAT3 Core Fragment of DNA Binding Domain

**Fig. 7:**
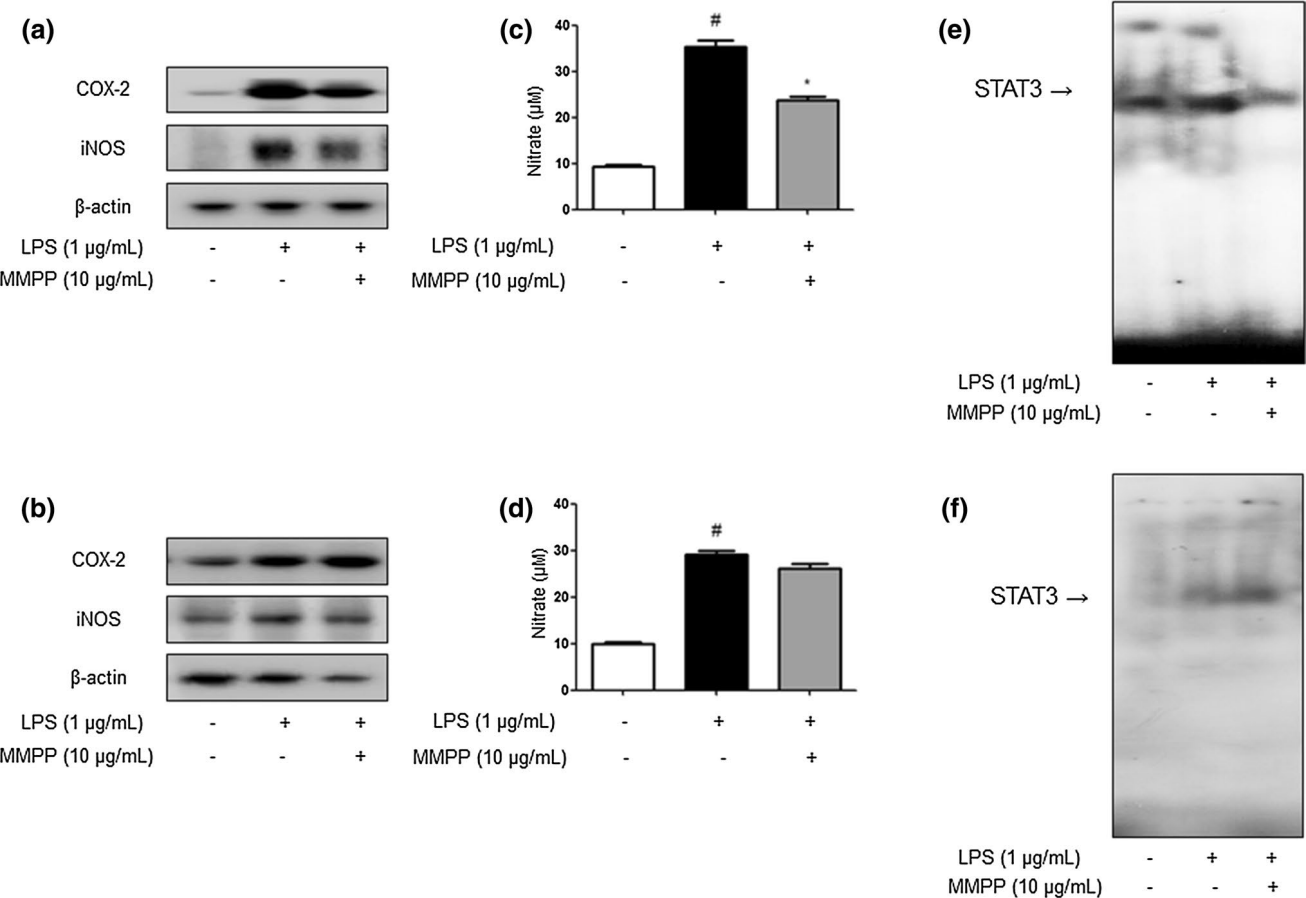
MMPP suppresses STAT3 activation and inflammatory protein expression through direct interaction with core fragment of STAT3 in LPS-stimulated RAW 264.7 cells. The effects of MMPP on the STAT3 activation and proinflammatory protein expression in LPS-stimulated murine macrophages were evaluated. Cells were transfected with a mutant STAT3 plasmid (T456A) followed by MMPP (10.0 μg/mL) treatment for 24 h, then effect of MMPP on the LPS-induced iNOS and COX-2 expression (**b**), NO production (**d**) and STAT3 DNA binding activity (**f**) were detected in LPS-stimulated cells. LPS-induced iNOS and COX-2 expression (**a**), NO production (**c**) and STAT3 DNA binding activity (**e**) of non-mutant form were also detected. Further, ^#^ *P* < 0.05 versus control, and *P < 0.05 versus LPS-treated control as determined by Student’s *t* test. Values are represented of three experiments. For the cropped images, samples were run in the same gels under same experimental conditions and processed in parallel

To further demonstrate the significant interaction of MMPP to STAT3, we investigated the inhibitory effect of MMPP on LPS-induced inflammatory responses. We treated MMPP (10.0 μg/mL) on LPS-treated macrophages cell line Raw 264.7 cells transiently transfected with the mutant of DNA binding domain of STAT3 (T456A) plasmid. We found that the inhibitory effect of MMPP on COX-2 and iNOS expression (Fig. 7b), NO generation (Fig. 7d) and STAT3 DNA binding activity (Fig. 7f) were diminished in the transient transfected Raw 264.7 cells. We also performed Western blot (Fig. 7a), NO assay (Fig. 7c) and EMSA (Fig. 7e) in non-transfected form of LPS-treated macrophages for comparing with STAT3 mutant form.

## Discussion

Accumulating epidemiological studies have suggested that neuroinflammatory responses may contribute to the progression of AD (Eikelenboom et al. 2002). The pathological hallmarks of neuroinflammation including marked astrogliosis, elevated release of proinflammatory mediators and cytokines appear in AD patients (Lee et al. 2012a). Our and other studies demonstrated that systemic injections of LPS-induced memory impairment through progressive neuroinflammation and amyloidogenesis (Lee et al. 2008; Lim et al. 2000; Hwang et al. 2016). We have demonstrated that several anti-inflammatory compounds such as 4-O-methylhonokiol, thiacremonone, obovatol and 2,4-bis(p-hydroxyphenyl)-2-butenal improved memory functions in AD animal models (Choi et al. 2012; Jin et al. 2013; Lee et al. 2012b; Yun et al. 2016).

In our recent study, we found that MMPP has strongest STAT3 DNA binding activities by luciferase activity in Raw 264.7 cells, and strongest binding affinity (−8.2 kcal/mol). Moreover, MMPP does not show any toxicities with suitable drug-likeness properties evaluated by computational ADME QSAR models using preAPMET and StarDrop soft program. MMPP exhibits significantly advantageous drug-likeness. (E)-2,4-bis(*p*-hydroxyphenyl)-2-butenal showed plausible mutagenicity in vivo and chromosome damage in vitro, while MMPP did not exhibit any of these mutagenic effects. (E)-2,4-bis(*p*-hydroxyphenyl)-2-butenal tested negative for other predicted toxicities except hepatotoxicity. However, MMPP treatment (5 mg/kg) for 3 weeks did not show hepatotoxic effects and MMPP showed better aqueous solubility, human intestinal absorption, and skin permeability. Furthermore, MMPP has multiple pharmacological properties against arthritis, neuroinflammation, and tumor growth, thus it is possible that MMPP could be a candidate compound to be developed as a drug (Zheng et al. 2017; Park et al. 2017).

It is notably reported that elevation of STAT3 phosphorylation is evident in postmortem samples of AD brains (Wan et al. 2010). STAT3 activates the transcription of BACE1, APP, presenilin-1 and γ-secretase and therefore increases production of Aβ_1-42_ because STAT3 transcriptionally regulates the mRNA levels of both BACE1 and presenilin-1 (Chiba et al. 2009; Liu et al. 2013). It was also reported that BACE1 transcription can be increased by inflammatory responses with activation of astrocytes and microglia induced by LPS as well as Aβ_1-42_ (Sambamurti et al. 2004). It is noteworthy that LPS could upregulate neuroinflammation in astrocytes and microglia through activation of STAT3. Activated astrocytes and microglia are closely associated with amyloid plaques in AD. Additionally, IL-6 and IL-10 are known for signaling via activation of transcription factor STAT3 (Niemand et al. 2003). It was also reported that increased IL-6 brain showed that severe cognitive impairments and behavioral deficits with neuronal loss (Wei et al. 2012). Furthermore, STAT3 has central roles in anti-inflammatory pathway, mediating IL-10 signals. These findings indicate that strategies to reduce IL-6 and IL-10 production may be particularly valuable for protecting the CNS disorders including AD. Our results indicate that MMPP could be a potential drug to control STAT3 activation and decrease production of neuroinflammatory cytokines including IL-6 and IL-10. Thus, inhibitory effects of MMPP on neuroinflammation and amyloidogenesis could be associated with its memory recovery effect, and targeting STAT3 is significantly contributing to these benefits. We previously found that MMPP directly binds to the DNA binding domain of STAT3 demonstrated by docking model, pull down assay as well as surface plasmon resonance (SPR) analysis. Moreover, we verified that the inhibitory effect of MMPP was abolished in the macrophages transiently transfected DNA binding domain mutant (T456A) plasmid. These data further demonstrated the significance of STAT3 in the biological effects of MMPP. Similar to our present data, other studies, as well as ours, have demonstrated that several compounds such as *ent*-Sauchinone (Song et al. 2014), 2,4-bis(4-hydroxyphenyl)-2-butenal diacetate (Kim et al. 2014), Kaempferol Glycosides (Yu et al. 2013) and Curcumin (Zhu et al. 2014) targeting STAT3 could be significant drugs for anti-amyloidogenesis and anti-inflammatory responses controlling the memory functions.

Dopenazil (Aricept^®^), Galantamine (Razadyne^®^) and Rivastigmine (Exelon^®^) are approved by the Food and Drug Administration (FDA) for treatment of AD as inhibiting the degradation of the neurotransmitter acetylcholine (Ach) (Pettenati et al. 2003; Farrimond et al. 2012). However, there are a number of AD patients undergoing various side effects by taking these drugs. For example, patients who are taking Aricept that overdose have experienced nausea, diarrhea, insomnia, and vomiting with gastrointestinal disorder. Furthermore, although AChE inhibitors help alleviate AD symptoms, they do not delay disease progression. Therefore, new therapeutic agents that block the disease-inducing mechanisms are essential (Yoo and Park 2012). Even though the mechanism of MMPP is different from FDA approved drugs, the improved toxicology of MMPP could be a promising candidate for treatment of AD. In addition, a combination of acetylcholinesterase inhibitor (AChEI) and inactivating the STAT3 pathway with MMPP treatment is a therapy of greater benefit in AD patients than taking AChEIs alone (Parsons et al. 2013; Park et al. 2013).

In conclusion, our findings demonstrate that MMPP has a potent therapeutic ability by inactivating STAT3 in LPS-treated mice AD model through inhibition of STAT3, which could result in the inhibition of Aβ accumulation by attenuating β-secretase activity. These findings suggest that one mechanism by which MMPP prevents anti-amyloidogenesis is due to a decrease in phosphorylation of STAT3. Thus, we suggest that MMPP could be useful for the treatment and/or prevention of AD.

## Electronic supplementary material

Below is the link to the electronic supplementary material.

Supplementary material 1 (TIFF 3073 kb)

## References

[CR1] Ban JO, Kim DH, Lee HP, Hwang CJ, Shim JH, Kim DJ (2014). Anti-arthritis effects of (E)-2,4-bis(p-hydroxyphenyl)-2-butenal are mediated by inhibition of the STAT3 pathway. British Journal of Pharmacology.

[CR2] Biesmans S, Meert TF, Bouwknecht JA, Acton PD, Davoodi N, De Haes P (2013). Systemic immune activation leads to neuroinflammation and sickness behavior in mice. Mediators of Inflammation.

[CR3] Carret-Rebillat AS, Pace C, Gourmaud S, Ravasi L, Montagne-Stora S, Longueville S (2015). Neuroinflammation and Abeta accumulation linked to systemic inflammation are decreased by genetic PKR down-regulation. Scientific Reports.

[CR4] Chen J, Buchanan JB, Sparkman NL, Godbout JP, Freund GG, Johnson RW (2008). Neuroinflammation and disruption in working memory in aged mice after acute stimulation of the peripheral innate immune system. Brain, Behavior, and Immunity.

[CR5] Cherubini E, Miles R (2015). The CA3 region of the hippocampus: how is it? What is it for? How does it do it?. Frontiers in Cellular Neuroscience.

[CR6] Chiba T, Yamada M, Aiso S (2009). Targeting the JAK2/STAT3 axis in Alzheimer’s disease. Expert Opinion on Therapeutic Targets.

[CR7] Cho SH, Park MH, Lee HP, Back MK, Sung HC, Chang HW (2014). (E)-2,4-Bis(p-hydroxyphenyl)-2-butenal enhanced TRAIL-induced apoptosis in ovarian cancer cells through downregulation of NF-kappaB/STAT3 pathway. Archives of Pharmacal Research.

[CR8] Choi DY, Lee JW, Lin G, Lee YK, Lee YH, Choi IS (2012). Obovatol attenuates LPS-induced memory impairments in mice via inhibition of NF-kappaB signaling pathway. Neurochemistry International.

[CR9] Cotter DR, Pariante CM, Everall IP (2001). Glial cell abnormalities in major psychiatric disorders: The evidence and implications. Brain Research Bulletin.

[CR10] DiCarlo G, Wilcock D, Henderson D, Gordon M, Morgan D (2001). Intrahippocampal LPS injections reduce Abeta load in APP + PS1 transgenic mice. Neurobiology of Aging.

[CR11] Eikelenboom P, Bate C, Van Gool WA, Hoozemans JJ, Rozemuller JM, Veerhuis R (2002). Neuroinflammation in Alzheimer’s disease and prion disease. Glia.

[CR12] Fan L, Wang T, Chang L, Song Y, Wu Y, Ma D (2014). Systemic inflammation induces a profound long term brain cell injury in rats. Acta Neurobiologiae Expermentalis (Wars).

[CR13] Farrimond LE, Roberts E, McShane R (2012). Memantine and cholinesterase inhibitor combination therapy for Alzheimer’s disease: A systematic review. British Medical Journal Open.

[CR14] Gu SM, Park MH, Hwang CJ, Song HS, Lee US, Han SB (2015). Bee venom ameliorates lipopolysaccharide-induced memory loss by preventing NF-kappaB pathway. Journal of Neuroinflammation.

[CR15] Hashimoto Y, Suzuki H, Aiso S, Niikura T, Nishimoto I, Matsuoka M (2005). Involvement of tyrosine kinases and STAT3 in Humanin-mediated neuroprotection. Life Sciences.

[CR16] Hauss-Wegrzyniak B, Lukovic L, Bigaud M, Stoeckel ME (1998). Brain inflammatory response induced by intracerebroventricular infusion of lipopolysaccharide: An immunohistochemical study. Brain Research.

[CR17] Henry CJ, Huang Y, Wynne AM, Godbout JP (2009). Peripheral lipopolysaccharide (LPS) challenge promotes microglial hyperactivity in aged mice that is associated with exaggerated induction of both pro-inflammatory IL-1beta and anti-inflammatory IL-10 cytokines. Brain, Behavior, and Immunity.

[CR18] Holsinger RM, McLean CA, Beyreuther K, Masters CL, Evin G (2002). Increased expression of the amyloid precursor beta-secretase in Alzheimer’s disease. Annals of Neurology.

[CR19] Hwang CJ, Park MH, Hwang JY, Kim JH, Yun NY, Oh SY (2016). CCR5 deficiency accelerates lipopolysaccharide-induced astrogliosis, amyloid-beta deposit and impaired memory function. Oncotarget.

[CR20] Jin P, Kim JA, Choi DY, Lee YJ, Jung HS, Hong JT (2013). Anti-inflammatory and anti-amyloidogenic effects of a small molecule, 2,4-bis(p-hydroxyphenyl)-2-butenal in Tg2576 Alzheimer’s disease mice model. Journal of Neuroinflammation.

[CR21] Kesner RP (2007). Behavioral functions of the CA3 subregion of the hippocampus. Learning & Memory.

[CR22] Kim JA, Yun HM, Jin P, Lee HP, Han JY, Udumula V (2014). Inhibitory effect of a 2,4-bis(4-hydroxyphenyl)-2-butenal diacetate on neuro-inflammatory reactions via inhibition of STAT1 and STAT3 activation in cultured astrocytes and microglial BV-2 cells. Neuropharmacology.

[CR23] Korolainen MA, Auriola S, Nyman TA, Alafuzoff I, Pirttila T (2005). Proteomic analysis of glial fibrillary acidic protein in Alzheimer’s disease and aging brain. Neurobiology of Diseases.

[CR24] Lee YJ, Choi DY, Choi IS, Kim KH, Kim YH, Kim HM (2012). Inhibitory effect of 4-O-methylhonokiol on lipopolysaccharide-induced neuroinflammation, amyloidogenesis and memory impairment via inhibition of nuclear factor-kappaB in vitro and in vivo models. Journal of Neuroinflammation.

[CR25] Lee YJ, Choi DY, Lee YK, Lee YM, Han SB, Kim YH (2012). 4-O-methylhonokiol prevents memory impairment in the Tg2576 transgenic mice model of Alzheimer’s disease via regulation of beta-secretase activity. Journal of Alzheimer’s Disease.

[CR26] Lee YJ, Choi IS, Park MH, Lee YM, Song JK, Kim YH (2011). 4-O-Methylhonokiol attenuates memory impairment in presenilin 2 mutant mice through reduction of oxidative damage and inactivation of astrocytes and the ERK pathway. Free Radical Biology and Medicine.

[CR27] Lee YJ, Choi DY, Yun YP, Han SB, Kim HM, Lee K (2013). Ethanol extract of Magnolia officinalis prevents lipopolysaccharide-induced memory deficiency via its antineuroinflammatory and antiamyloidogenic effects. Phytotherapy Research.

[CR28] Lee YJ, Choi DY, Yun YP, Han SB, Oh KW, Hong JT (2013). Epigallocatechin-3-gallate prevents systemic inflammation-induced memory deficiency and amyloidogenesis via its anti-neuroinflammatory properties. Journal of Nutritional Biochemistry.

[CR29] Lee JW, Lee YK, Yuk DY, Choi DY, Ban SB, Oh KW (2008). Neuro-inflammation induced by lipopolysaccharide causes cognitive impairment through enhancement of beta-amyloid generation. Journal of Neuroinflammation.

[CR30] Lim GP, Yang F, Chu T, Chen P, Beech W, Teter B (2000). Ibuprofen suppresses plaque pathology and inflammation in a mouse model for Alzheimer’s disease. Journal of Neuroscience.

[CR31] Lisman JE (1999). Relating hippocampal circuitry to function: Recall of memory sequences by reciprocal dentate-CA3 interactions. Neuron.

[CR32] Liu L, Martin R, Kohler G, Chan C (2013). Palmitate induces transcriptional regulation of BACE1 and presenilin by STAT3 in neurons mediated by astrocytes. Experimental Neurology.

[CR33] Madeddu S, Woods TA, Mukherjee P, Sturdevant D, Butchi NB, Peterson KE (2015). Identification of Glial activation markers by comparison of transcriptome changes between astrocytes and microglia following innate immune stimulation. PLoS ONE.

[CR34] Madronal N, Delgado-Garcia JM, Fernandez-Guizan A, Chatterjee J, Kohn M, Mattucci C (2016). Rapid erasure of hippocampal memory following inhibition of dentate gyrus granule cells. Nature Communications.

[CR35] Masocha W (2009). Systemic lipopolysaccharide (LPS)-induced microglial activation results in different temporal reduction of CD200 and CD200 receptor gene expression in the brain. Journal of Neuroimmunology.

[CR37] Morris R (1984). Developments of a water-maze procedure for studying spatial learning in the rat. Journal of Neuroscience Methods.

[CR38] Niemand C, Nimmesgern A, Haan S, Fischer P, Schaper F, Rossaint R (2003). Activation of STAT3 by IL-6 and IL-10 in primary human macrophages is differentially modulated by suppressor of cytokine signaling 3. The Journal of Immunology.

[CR39] Noh H, Jeon J, Seo H (2014). Systemic injection of LPS induces region-specific neuroinflammation and mitochondrial dysfunction in normal mouse brain. Neurochemistry International.

[CR40] Park SJ, Shin EJ, Min SS, An J, Li Z, Hee Chung Y (2013). Inactivation of JAK2/STAT3 signaling axis and downregulation of M1 mAChR cause cognitive impairment in klotho mutant mice, a genetic model of aging. Neuropsychopharmacology.

[CR41] Park CW, Song YS, Lee HP, Hong JT, Yoon DY (2017). (E)-2-methoxy-4-(3-(4-methoxyphenyl)prop-1-en-1-yl)phenol induces apoptosis in HeLa cervical cancer cells via the extrinsic apoptotic pathway. Journal of Microbiology and Biotechnology.

[CR42] Parsons CG, Danysz W, Dekundy A, Pulte I (2013). Memantine and cholinesterase inhibitors: Complementary mechanisms in the treatment of Alzheimer’s disease. Neurotoxicity Research.

[CR43] Pettenati C, Annicchiarico R, Caltagirone C (2003). Clinical pharmacology of anti-Alzheimer drugs. Fundamental & Clinical Pharmacology.

[CR44] Pop V, Head E, Berchtold NC, Glabe CG, Studzinski CM, Weidner AM (2012). Abeta aggregation profiles and shifts in APP processing favor amyloidogenesis in canines. Neurobiology of Aging.

[CR45] Pulawski W, Ghoshdastider U, Andrisano V, Filipek S (2012). Ubiquitous amyloids. Applied Biochemistry and Biotechnology.

[CR46] Qin L, Wu X, Block ML, Liu Y, Breese GR, Hong JS (2007). Systemic LPS causes chronic neuroinflammation and progressive neurodegeneration. Glia.

[CR47] Sambamurti K, Kinsey R, Maloney B, Ge YW, Lahiri DK (2004). Gene structure and organization of the human beta-secretase (BACE) promoter. The FASEB Journal.

[CR48] Schnabel J (2010). Protein folding: The dark side of proteins. Nature.

[CR49] Sheng JG, Bora SH, Xu G, Borchelt DR, Price DL, Koliatsos VE (2003). Lipopolysaccharide-induced-neuroinflammation increases intracellular accumulation of amyloid precursor protein and amyloid beta peptide in APPswe transgenic mice. Neurobiology of Diseases.

[CR50] Sola C, Casal C, Tusell JM, Serratosa J (2002). Astrocytes enhance lipopolysaccharide-induced nitric oxide production by microglial cells. European Journal of Neuroscience.

[CR60] Son DJ, Kim DH, Nah SS, Park MH, Lee HP, Han SB (2016). Novel synthetic (E)-2-methoxy-4-(3-(4-methoxyphenyl) prop-1-en-1-yl) phenol inhibits arthritis by targeting signal transducer and activator of transcription 3. Scientific Reports.

[CR51] Song SY, Jung YY, Hwang CJ, Lee HP, Sok CH, Kim JH (2014). Inhibitory effect of ent-Sauchinone on amyloidogenesis via inhibition of STAT3-mediated NF-kappaB activation in cultured astrocytes and microglial BV-2 cells. Journal of Neuroinflammation.

[CR52] Wan J, Fu AK, Ip FC, Ng HK, Hugon J, Page G (2010). Tyk2/STAT3 signaling mediates beta-amyloid-induced neuronal cell death: implications in Alzheimer’s disease. Journal of Neuroscience.

[CR53] Wei H, Chadman KK, McCloskey DP, Sheikh AM, Malik M, Brown WT (2012). Brain IL-6 elevation causes neuronal circuitry imbalances and mediates autism-like behaviors. Biochimica et Biophysica Acta.

[CR54] Wen Y, Yu WH, Maloney B, Bailey J, Ma J, Marie I (2008). Transcriptional regulation of beta-secretase by p25/cdk5 leads to enhanced amyloidogenic processing. Neuron.

[CR55] Yoo KY, Park SY (2012). Terpenoids as potential anti-Alzheimer’s disease therapeutics. Molecules.

[CR56] Yu L, Chen C, Wang LF, Kuang X, Liu K, Zhang H (2013). Neuroprotective effect of kaempferol glycosides against brain injury and neuroinflammation by inhibiting the activation of NF-kappaB and STAT3 in transient focal stroke. PLoS ONE.

[CR57] Yun HM, Jin P, Park KR, Hwang J, Jeong HS, Kim EC (2016). Thiacremonone potentiates anti-oxidant effects to improve memory dysfunction in an APP/PS1 transgenic mice model. Molecular Neurobiology.

[CR58] Zheng J, Son DJ, Lee HL, Lee HP, Kim TH, Joo JH (2017). (E)-2-methoxy-4-(3-(4-methoxyphenyl)prop-1-en-1-yl)phenol suppresses ovarian cancer cell growth via inhibition of ERK and STAT3. Molecular Carcinogenesis.

[CR59] Zhu HT, Bian C, Yuan JC, Chu WH, Xiang X, Chen F (2014). Curcumin attenuates acute inflammatory injury by inhibiting the TLR4/MyD88/NF-kappaB signaling pathway in experimental traumatic brain injury. Journal of Neuroinflammation.

