# Supplementary figures and images for: (E)-2-Methoxy-4-(3-(4-methoxyphenyl) prop-1-en-1-yl) Phenol Ameliorates LPS-Mediated Memory Impairment by Inhibition of STAT3 Pathway
Source: Neuromolecular Med. 2017 Oct 19;19(4):555–70. doi: 10.1007/s12017-017-8469-3 (PMC5683055; doi:10.1007/s12017-017-8469-3)

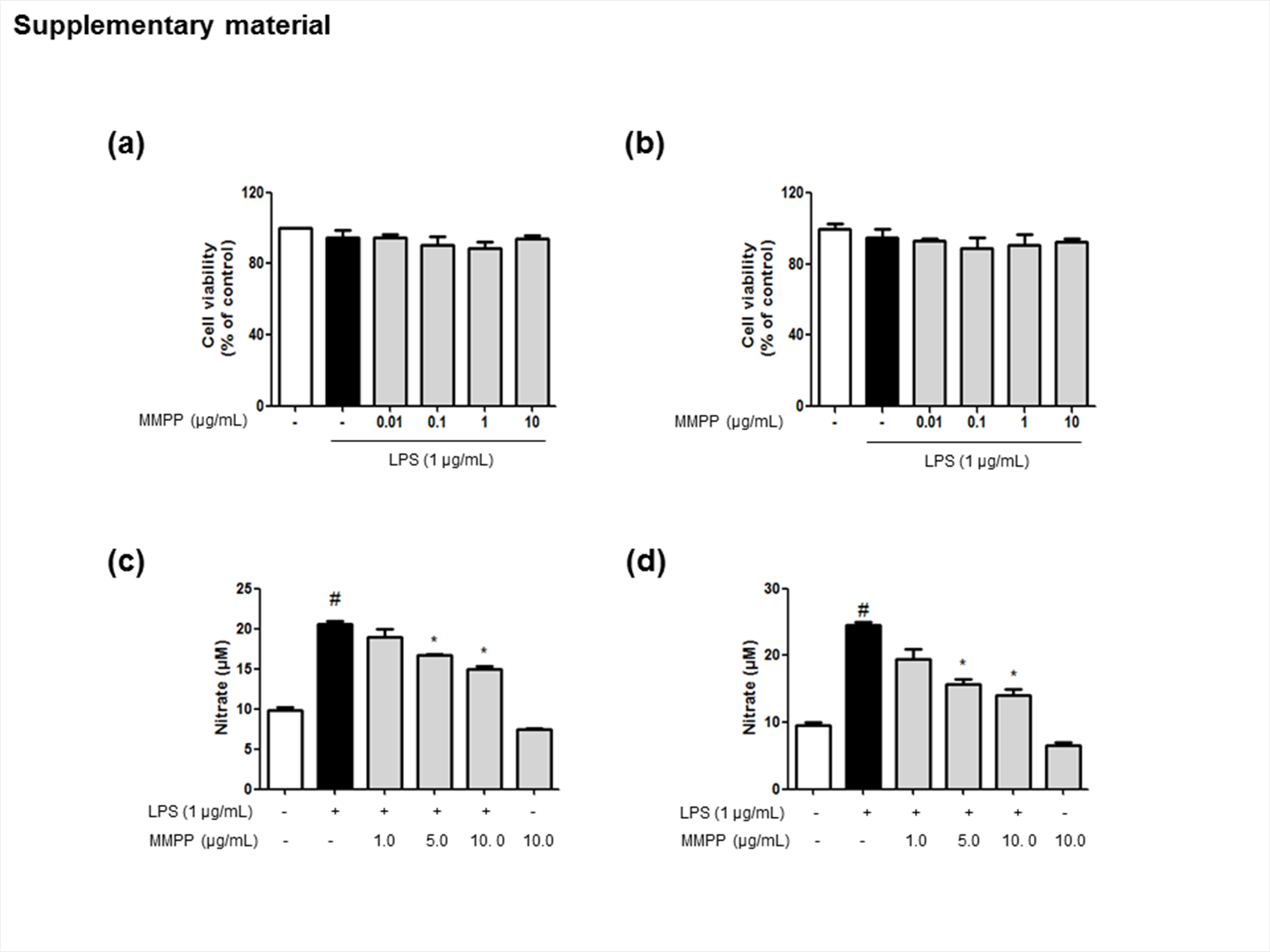

Supplement: Supplementary file 1 — Supplementary material 1 (TIFF 3073 kb) [file 12017_2017_8469_MOESM1_ESM.tif]
